# Supplementary material for: Three New Dihydrophenanthrene Derivatives from Cymbidium ensifolium and Their Cytotoxicity against Cancer Cells
Source: Molecules. 2022 Mar 29;27(7):2222. doi: 10.3390/molecules27072222 (PMC9000781; doi:10.3390/molecules27072222)
Supplement: Supplementary file 1 [file molecules-27-02222-s001.zip › molecules-1648131-supplementary.pdf]

SUPPLEMENTARY MATERIAL

Three new dihydrophenanthrene derivatives from *Cymbidium ensifolium* and their cytotoxicity against cancer cells

Jimoh Tajudeen Ogundare [1,2], Bruno Cesar Costa [1,3], Chaisak Chansriniyom [4,5], Chatchai Chaotham [3,6], Pithi Chanvorachote [6,7], Pornchai Rojsitthisak [8,9], Kittisak Likhitwitayawuid [4] and Boonchoo Sritularak [4,9,*]

[1] Pharmaceutical Sciences and Technology Program, Faculty of Pharmaceutical Sciences, Chulalongkorn University, Bangkok 10330, Thailand; jimmmypeace@gmail.com (J.T.O.); brunocesarsoares@outlook.com.br (B.C.C.)
[2] Department of Biochemistry, Faculty of Health Sciences, Islamic University in Uganda P. O. Box 7689, Kampala, Uganda
[3] Department of Biochemistry and Microbiology, Faculty of Pharmaceutical Sciences, Chulalongkorn University, Bangkok 10330, Thailand; cchoatham@gmail.com (C.C.)
[4] Department of Pharmacognosy and Pharmaceutical Botany, Faculty of Pharmaceutical Sciences, Chulalongkorn University, Bangkok 10330, Thailand; Chaisak.Ch@chula.ac.th (C.C.); Kittisak.L@chula.ac.th (K.L.)
[5] Natural products and Nanoparticles Research Unit, Chulalongkorn University, Bangkok, 10330
[6] Center of Excellence in Cancer Cell and Molecular Biology, Faculty of Pharmaceutical Sciences, Chulalongkorn University, Bangkok 10330, Thailand
[7] Department of Pharmacology and Physiology, Faculty of Pharmaceutical Sciences, Chulalongkorn University, Bangkok 10330, Thailand; pithi.c@chula.ac.th (P.C.)
[8] Department of Food and Pharmaceutical Chemistry, Faculty of Pharmaceutical Sciences, Chulalongkorn University, Bangkok 10330, Thailand; pornchai.r@chula.ac.th (P.R.)
[9] Center of Excellence in Natural Products for Ageing and Chronic Diseases, Faculty of Pharmaceutical Sciences, Chulalongkorn University, Bangkok, 10330, Thailand.
* Correspondence: boonchoo.sr@chula.ac.th (B.S.)

**Contents**	**page**
Figure S1. ^{1}H NMR spectrum of **1** (400 MHz) in acetone-d_6	3
Figure S2. ^{13}C NMR spectrum of **1** (100 MHz) in acetone-d_6	3
Figure S3. HSQC spectrum of **1** in acetone-d_6	4
Figure S4. HMBC spectrum of **1** in acetone-d_6	4
Figure S5. COSY spectrum of **1** in acetone-d_6	5
Figure S6. NOESY spectrum of **1** in acetone-d_6	5
Figure S7. HRESIMS spectrum of **1**	6

**Contents**	**page**
Figure S8. ^{1}H NMR spectrum of **2** (400 MHz) in acetone-d_6	6
Figure S9. ^{13}C NMR spectrum of **2** (100 MHz) in acetone-d_6	7
Figure S10. HSQC spectrum of **2** in acetone-d_6	7
Figure S11. HMBC spectrum of **2** in acetone-d_6	8
Figure S12. COSY spectrum of **2** in acetone-d_6	8
Figure S13. NOESY spectrum of **2** in acetone-d_6	9
Figure S14. HRESIMS spectrum of **2**	9
Figure S15. ^{1}H NMR spectrum of **3** (400 MHz) in acetone-d_6	10
Figure S16. ^{13}C NMR spectrum of **3** (100 MHz) in acetone-d_6	10
Figure S17. HSQC spectrum of **3** in acetone-d_6	11
Figure S18. HMBC spectrum of **3** in acetone-d_6	11
Figure S19. NOESY spectrum of **3** in acetone-d_6	12
Figure S20. HRESIMS spectrum of **3**	12

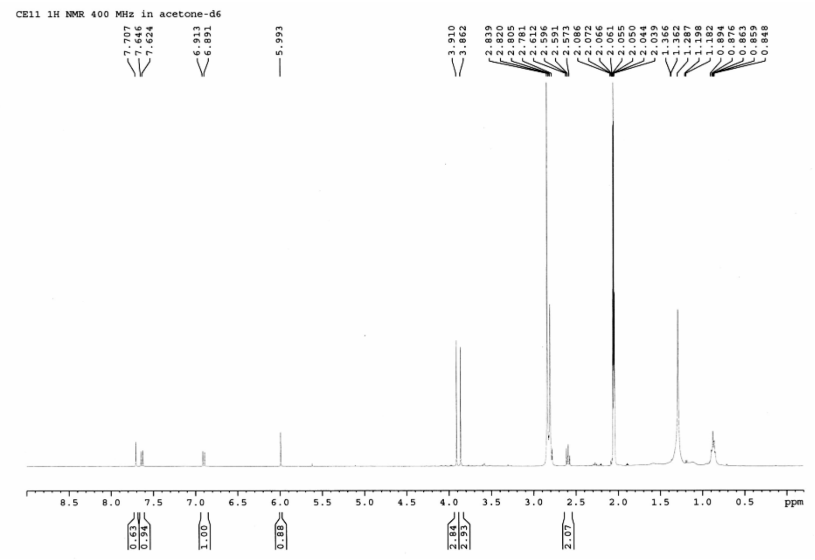

Figure S1. ^{1}H NMR spectrum of **1** (400 MHz) in acetone-d_6.

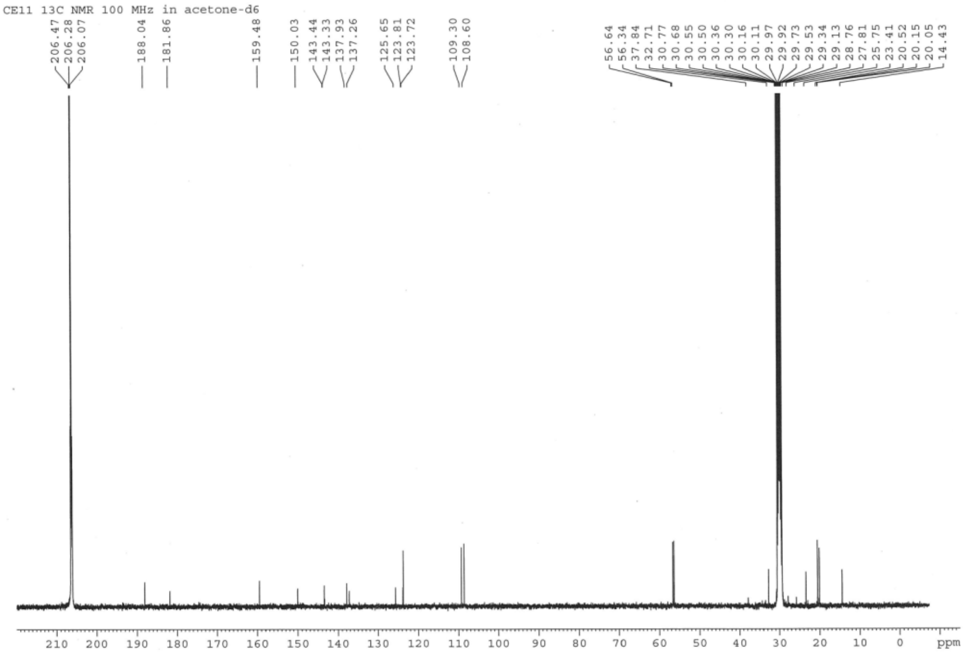

Figure S2. ^{13}C NMR spectrum of **1** (100 MHz) in acetone-d_6.

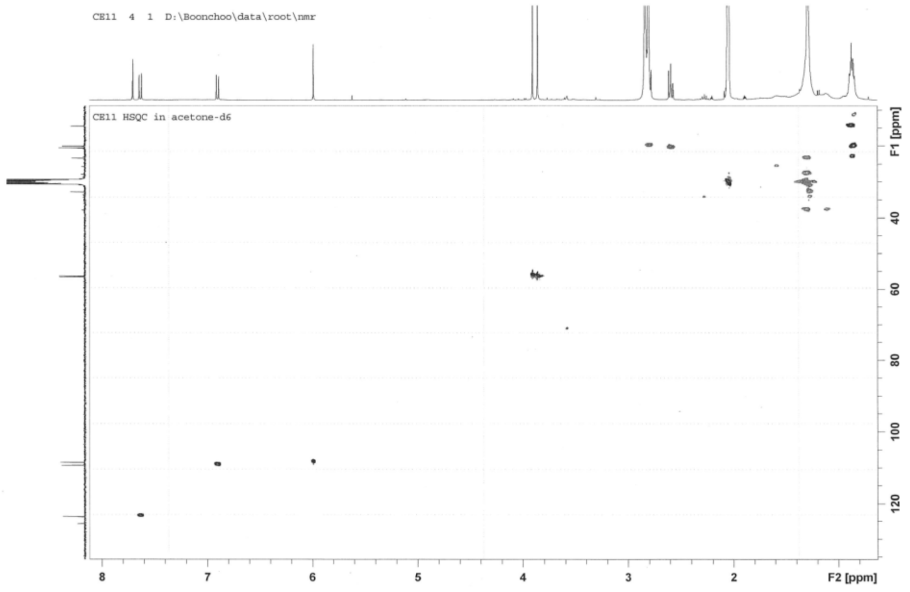

Figure S3. HSQC spectrum of **1** in acetone-d_6.

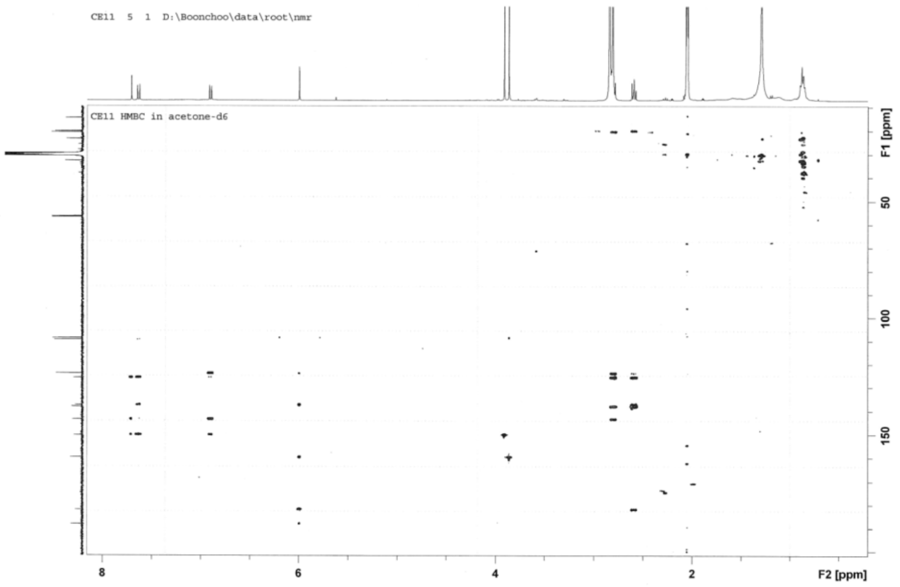

Figure S4. HMBC spectrum of **1** in acetone-d_6.

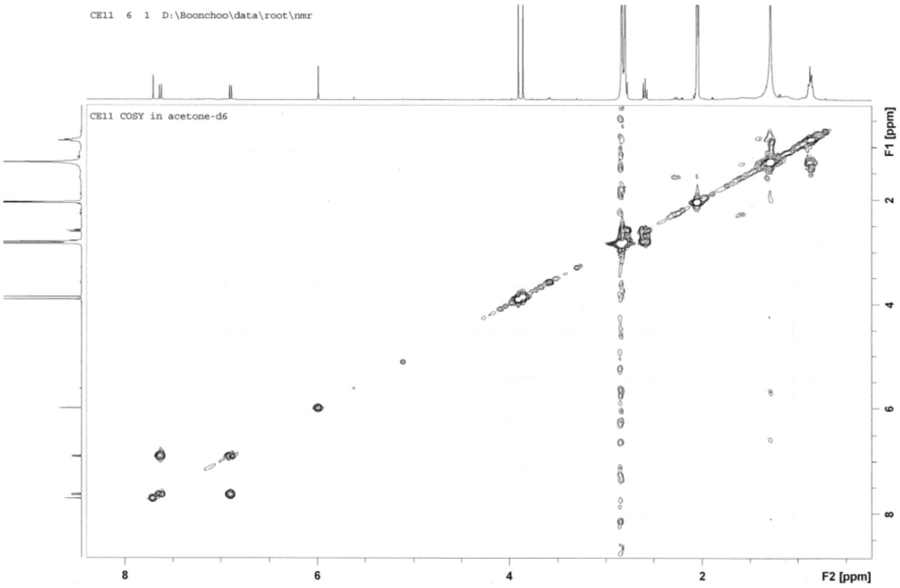

Figure S5. COSY spectrum of **1** in acetone-d_6.

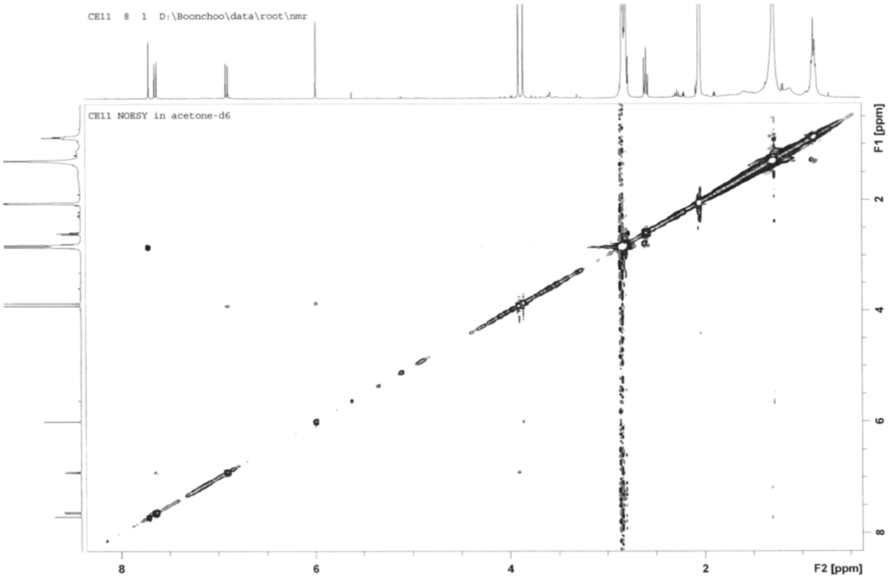

Figure S6. NOESY spectrum of **1** in acetone-d_6.

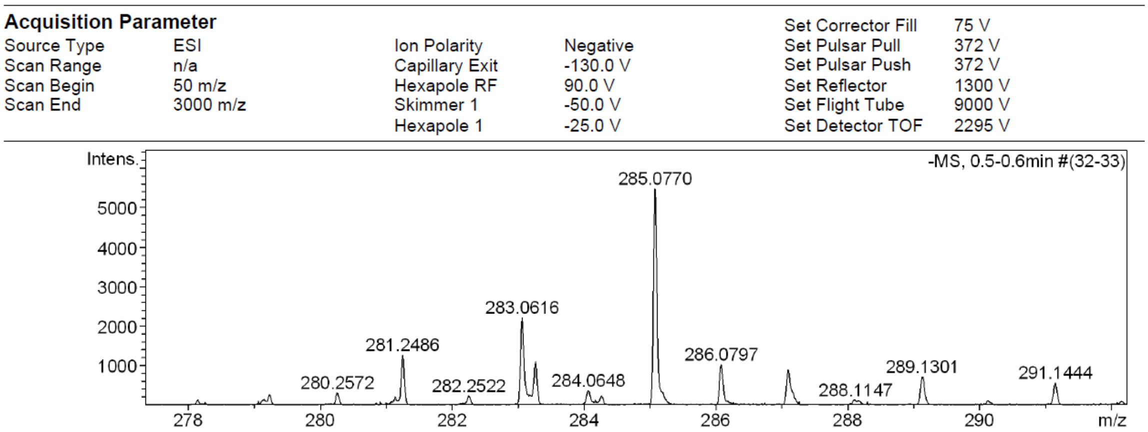

Figure S7. HRESIMS spectrum of **1**.

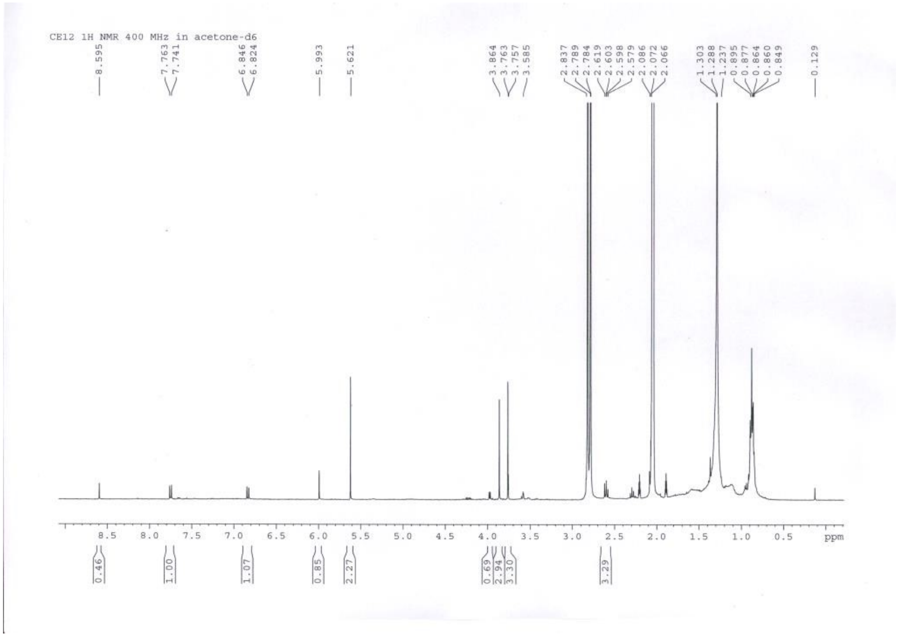

Figure S8. ^{1}H NMR spectrum of **2** (400 MHz) in acetone-d_6.

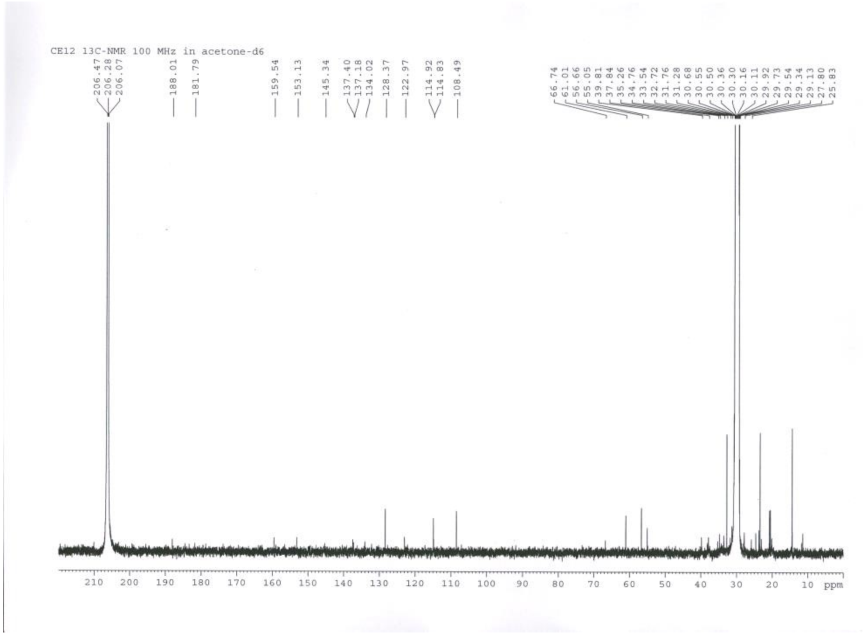

Figure S9. ^{13}C NMR spectrum of **2** (100 MHz) in acetone-d_6.

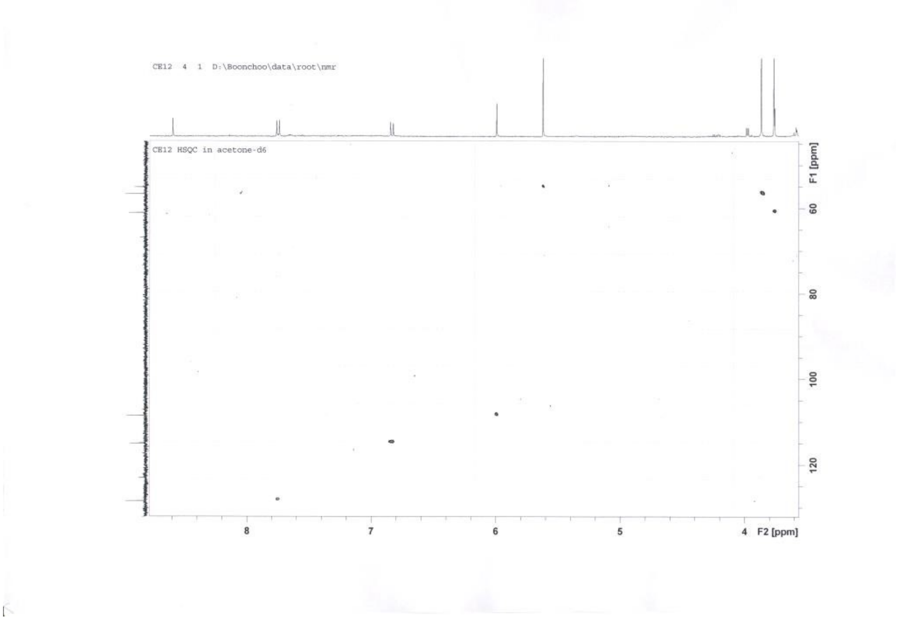

Figure S10. HSQC spectrum of **2** in acetone-d_6.

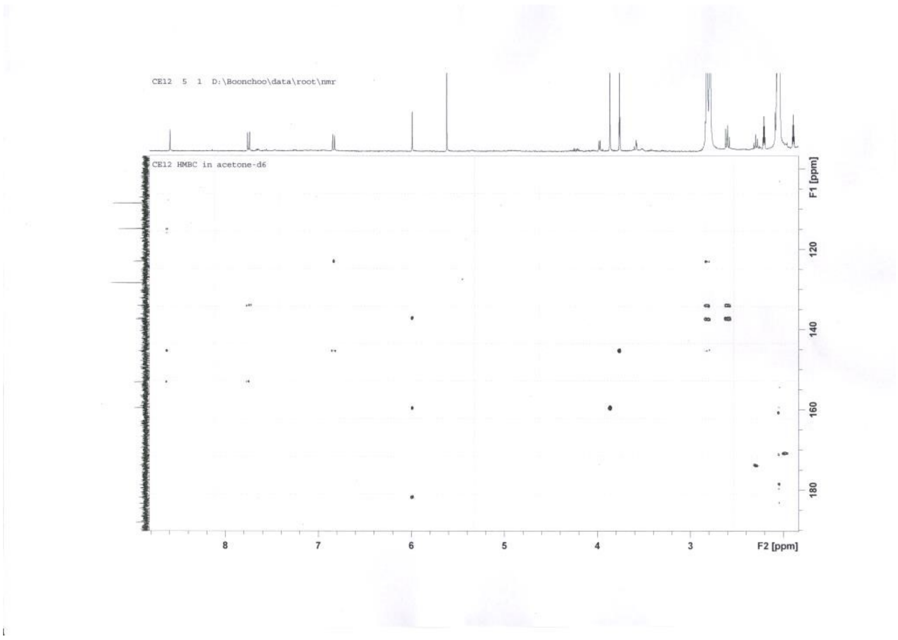

Figure S11. HMBC spectrum of **2** in acetone-d_6.

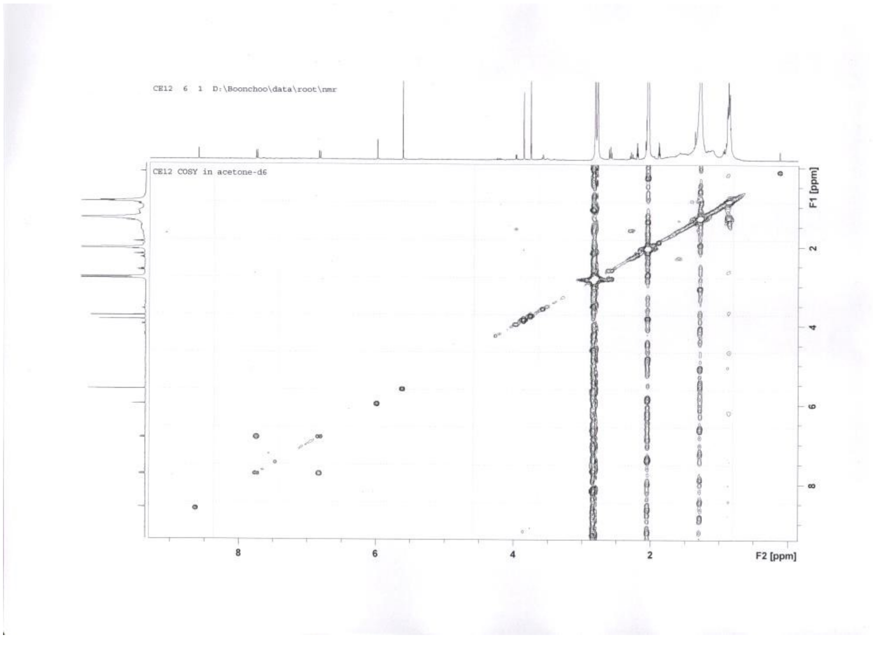

Figure S12. COSY spectrum of **2** in acetone-d_6.

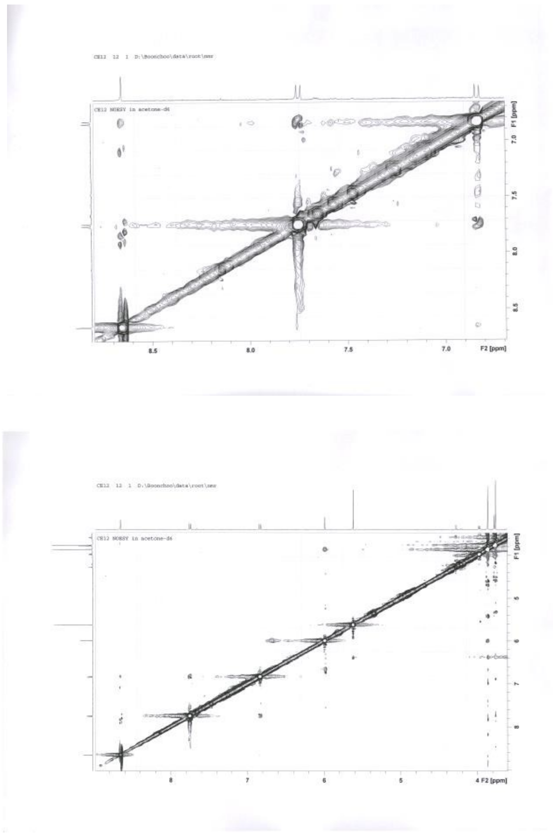

Figure S13. NOESY spectrum of **2** in acetone-d_6.

Acquisition Parameter					
				Set Corrector Fill	75 V
Source Type	ESI	Ion Polarity	Negative	Set Pulsar Pull	372 V
Scan Range	n/a	Capillary Exit	-60.0 V	Set Pulsar Push	372 V
Scan Begin	50 m/z	Hexapole RF	90.0 V	Set Reflector	1300 V
Scan End	3000 m/z	Skimmer 1	-50.0 V	Set Flight Tube	9000 V
		Hexapole 1	-25.0 V	Set Detector TOF	2295 V

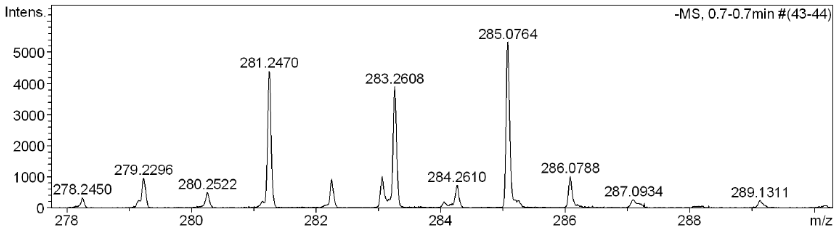

Figure S14. HRESIMS spectrum of **2**.

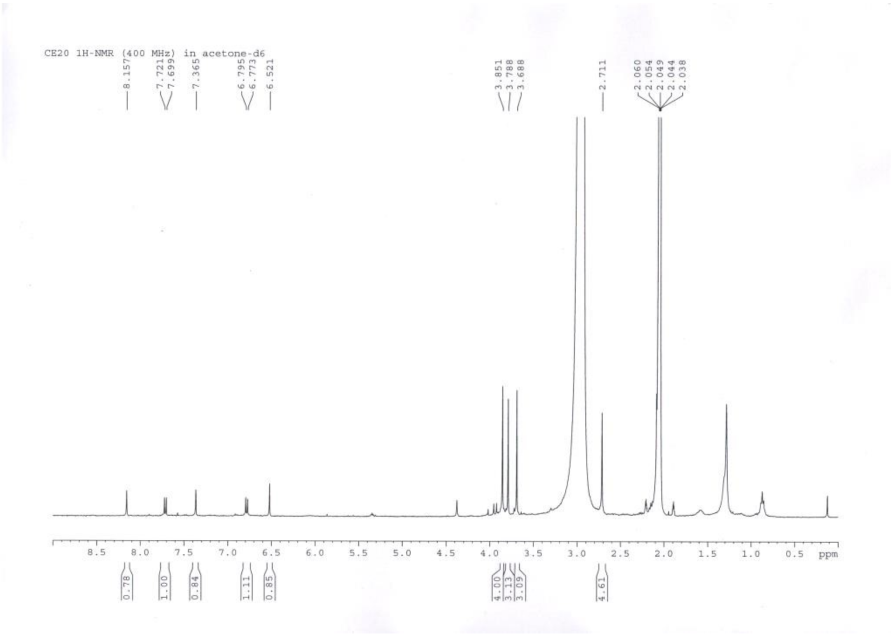

Figure S15. ^{1}H NMR spectrum of **3** (400 MHz) in acetone-*d*$_6$.

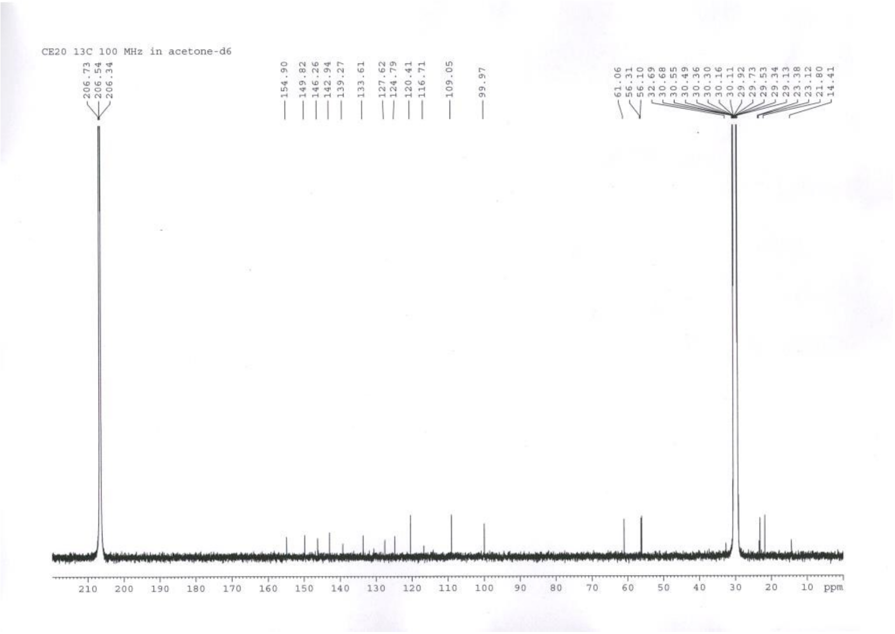

Figure S16. ^{13}C NMR spectrum of **3** (100 MHz) in acetone-*d*$_6$.

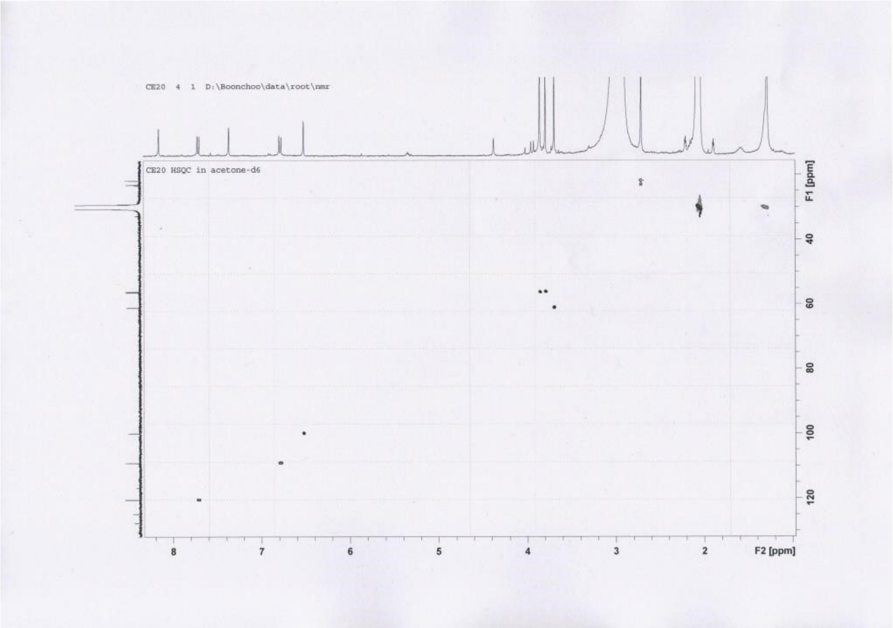

Figure S17. HSQC spectrum of **3** in acetone-d_6.

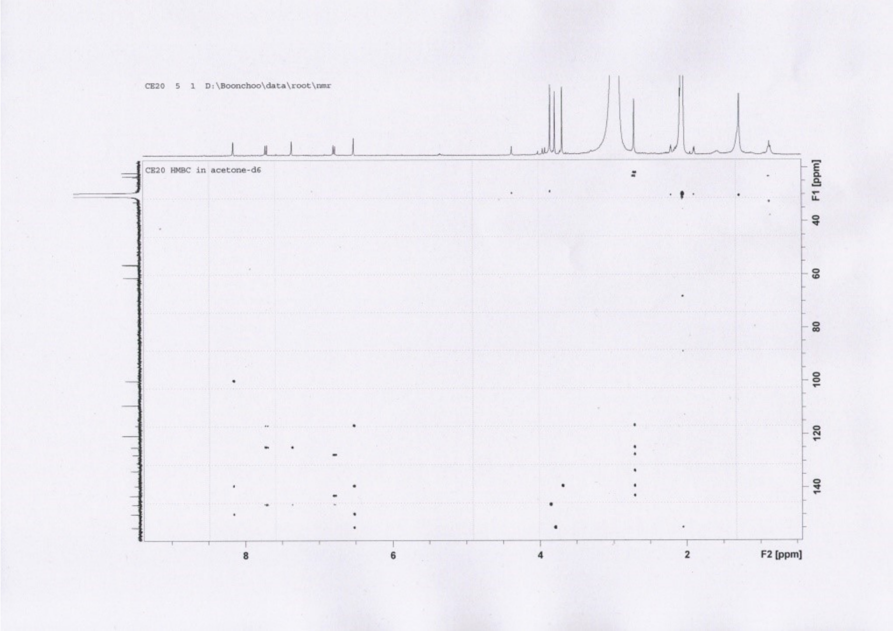

Figure S18. HMBC spectrum of **3** in acetone-d_6.

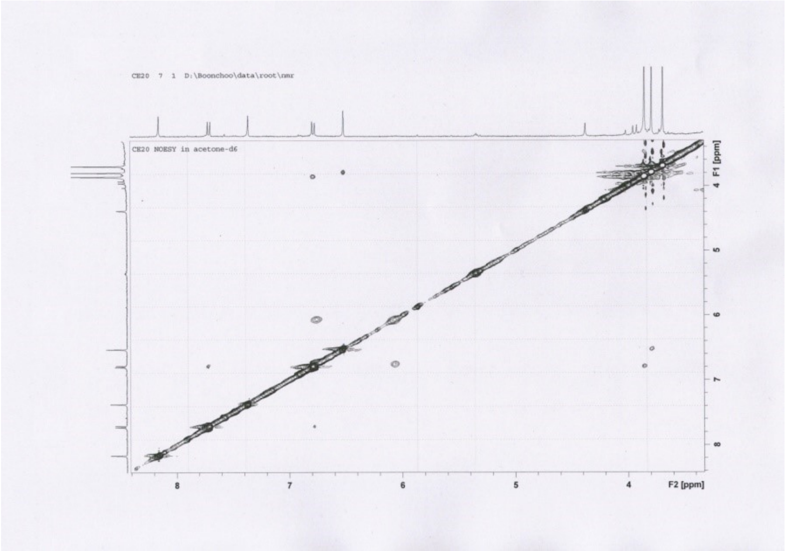

Figure S19. NOESY spectrum of **3** in acetone-d_6.

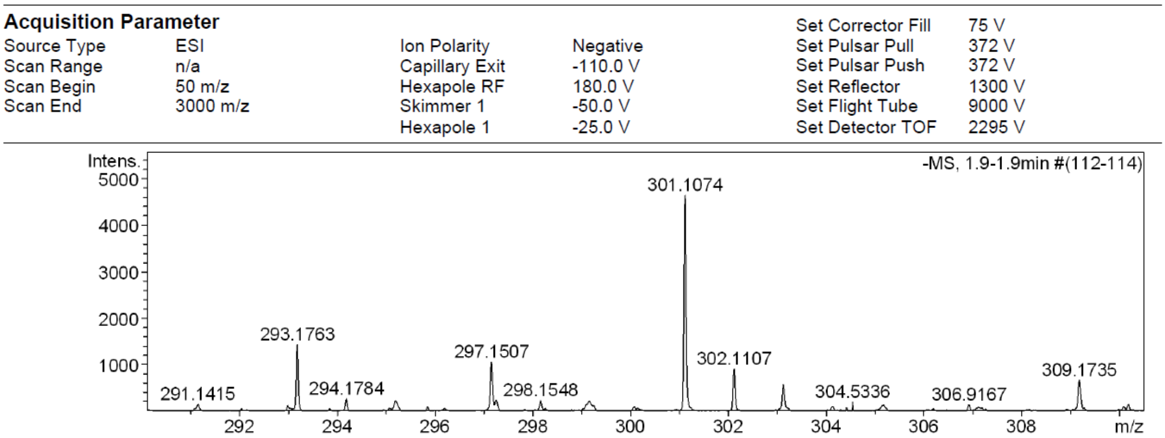

Figure S20. HRESIMS spectrum of **3**.
